# Modelling life course blood pressure trajectories using Bayesian adaptive splines

**DOI:** 10.1177/0962280214532576

**Published:** 2014-04-25

**Authors:** Graciela Muniz-Terrera, Eleni Bakra, Rebecca Hardy, Fiona E Matthews, David Lunn, FALCon collaboration group

**Affiliations:** 1 MRC Unit for Lifelong Health and Ageing at UCL, London, UK; 2 MRC Biostatistics Unit

**Keywords:** adaptive Bayesian splines, repeated measurements, hierarchical models, spline regression, reversible jump Markov chain Monte Carlo, blood pressure

## Abstract

No single study has collected data over individuals’ entire lifespans. To understand changes over the entire life course, it is necessary to combine data from various studies that cover the whole life course. Such combination may be methodologically challenging due to potential differences in study protocols, information available and instruments used to measure the outcome of interest. Motivated by our interest in modelling blood pressure changes over the life course, we propose the use of Bayesian adaptive splines within a hierarchical setting to combine data from several UK-based longitudinal studies where blood pressure measures were taken in different stages of life. Our method allowed us to obtain a realistic estimate of the mean life course trajectory, quantify the variability both within and between studies, and examine overall and study specific effects of relevant risk factors on life course blood pressure changes.

## 1 Introduction

Most evidence about changes over the lifespan in markers of physical function has been produced either by comparison of cross sectional data at different points in time or by longitudinal studies with limited follow-up. The comparison of cross sectional data can produce misleading results as samples compared over time may differ due to differential dropout, study design, cohort effects and eventual changes in measuring instruments. Although some of these are factors that also affect longitudinal studies, longitudinal data offer the opportunity of investigating population and person level changes in the outcome of interest. Yet, most longitudinal studies conducted so far have limited follow-up. Although studies exist (1946 British birth cohort, 1958 British birth cohort) where individuals have been followed since birth, study participants have not yet reached old age. Therefore, to describe changes over the entire lifespan, it is necessary to combine data from studies where individuals have been assessed during different stages of life.

The combination of data from longitudinal studies, as of cross sectional studies, is methodologically challenging. As mentioned before, studies will be likely to differ in their design and these differences will need to be taken into account in the models. For example, some samples may be age homogeneous at study entry, whilst other samples may be conformed by an age heterogeneous group; some samples may be population representative whilst others may be selected samples; and some studies may be gender specific whilst other studies may include both men and women. It is likely that in some situations, studies may have employed different instruments to measure the same variable of interest. Furthermore, these differences may be present within the same study when instruments were changed in the different data collection waves. For instance, as more technologically advanced devices became available, a switch in the measuring device used to measure blood pressure occurred between the 1982 and 1989 assessments in the NSHD 1946.

Longitudinal data are often modelled using parametric random effects models,^1^ as these models permit the description of mean change whilst informing about variability across individuals about that mean change. However, standard parametric formulations of random effects models may be inappropriate for the description of change of some biological markers as most of these parametric formulations produce unrealistic trajectory shapes.^2–4^ Non-parametric formulations of random effects models are a more flexible alternative for the description of change of biological markers. In particular, splines^5^ are an appealing and computationally efficient alternative that can be used to smooth noisy data. Initially an approach where a spline with a fixed and predetermined number of knots could be used to model the data; however, the choice and location of these knots may influence the estimation of the smoothed line. For example, a large number of knots would produce a very detailed line whilst a small number of knots may not capture the pattern of change sufficiently well.

To overcome these difficulties, DiMatteo et al.^6^ proposed the use of Bayesian adaptive splines to fit curves with free knots to data drawn from an exponential family. In DiMatteo’s proposed method, reversible jump Markov chain Monte Carlo (RJMCMC^7^) allows the *number* of knots, as well as their locations, to be estimated as model parameters; hence the spline’s level of detail adapts to the information present in the data. A prior distribution is required for the number of knots, their locations, the corresponding spline coefficients and the residual standard deviation. DiMatteo et al.’s chosen prior allowed the joint marginal posterior distribution for the number and location of knots to be obtained analytically (by integrating out the other parameters), allowing for efficient exploration of that marginal posterior by the RJMCMC method. Note that RJMCMC can be difficult to implement and often relies on the model being mathematically tractable in some way. DiMatteo et al. applied their approach to single studies, but it is not applicable in hierarchical settings, as are discussed in this paper.

In this paper, motivated by our interest in understanding changes in BP across the life course, we extend DiMatteo et al.’s method by incorporating a hierarchical structure to combine data from multiple longitudinal studies thus covering the entire life course. To the best of our knowledge, ours is the first attempt to model trajectories of a biomarker over the entire life course. Previously, Wills et al.^8^ modelled blood pressure measures of a set of longitudinal studies that include the studies used in our work independently using splines. In this publication, random effects models that described change using cubic and quadratic polynomials were fitted to blood pressure measures and the effect of several risk factors was examined. This paper produced relevant information about study-specific blood pressure changes and the effect of risk factors on each of these studies, but did not combine the different studies. This limited the ability of researchers to examine information such as variability across the different studies and of estimating a life course trajectory.

The paper is organised as follows. In Section 2, we describe the data sets used. Then, we describe the model structure in Section 3 and present results in Section 4. We discuss results in Section 5 and provide the WinBUGS code used for the analyses conducted in Appendix 1.

## 2 Data

Blood pressure measures from four studies were analysed here. These studies are part of the FALCon collaboration and were selected on the basis of being funded by the UK Medical Research Council and covering different but overlapping periods of the life course with at least two measures of blood pressure. Studies included in the analysis are: (1) the Avon Longitudinal Study of Parents And Children (ALSPAC), relating to children born in Avon in the 1990s;^9^ (2) the Medical Research Council (MRC) National Survey of Health and Development (NSHD)^10^ of individuals born in England, Scotland or Wales in 1946; (3) the Caerphilly Prospective Study (CAPS),^11^ which includes only men initially recruited in and (4) the Twenty-07 study (T-07),^12^ undertaken in the west of Scotland and started in 1986. The T-07 study is formed by three age defined subcohorts of individuals born in 1970s, 1950s and 1930s that were regarded as independent studies.

Blood pressure measures were taken by trained nurses in all studies except for CAPS, where measures were taken by physicians in the first four data collection waves and by a trained field worker in the fifth wave. In most studies at least two measurements were taken at each assessment. Instead, in CAPS one single blood pressure measure was taken in all collection waves except the second when two measures were taken. At the time of taking the measures, participants were seated and allowed at least 2 min rest prior to measurement. Different devices were used over time and in the different studies to take blood pressure reading. In ALSPAC and late waves of CAPS, NSHD and T-07 an automated oscillometric (AO) device was used to take the readings, whilst a manual random zero sphygmomanometer (MRZ) was used in earlier waves of the CAPS, NSHD and T-07 studies. Specifically, in CAPS, an MRZ machine was used in the first four waves and an AO machine in the last wave. In NSHD, an MRZ machine was used in the first two waves and an AO in the third one, whilst T-07 switched from an MRZ to an AO in the third wave, when both machines were used.

Table 1 presents the range of ages of individuals, the number of waves and total number of individuals for each cohort. Each study collected information specific to their original research aims, but a common core of socio-demographic variables such as participants’ height and weight, occupational social class at study entry and marital status was collected in all studies. In studies of young people (ALSPAC and youngest cohort of the T-07 study), social class was defined by the parents’ main occupation. Adult males’ and non-married women’s social classes were defined based on their own occupations; married women’s social classes were defined according to their husbands’ occupations or, if this information was not available, on their own occupations. Information about medication intake collected by nurses was available in all studies of individuals aged 18 years and older.

**Table 1.** Age range of individuals and number of waves within each cohort.

Study	Year initiation	Age range of participants	Number of data waves	Total number of individuals
ALSPAC	1990–1992	7–18	6	9432
T-07 (1970s)	1986	15–38	5	1481
T-07 (1950s)	1986	30–63	5	1391
T-07 (1930s)	1986	55–79	5	1479
NSHD	1946	36, 43, 53	3	3655
CAPS	1990–1992	43–85	5	2949

We derived a set of variables to account for factors known to be associated with hypertension. First, using information about social class, we derived an indicator of manual occupation at study entry. Body mass index (BMI) was calculated as weight/height2(kg/m2). Using published cut-off points,^13^ we defined binary indicators to classify individuals as underweight, normal weight and overweight.^14^ For children we used a different criterion as BMI classification varies by age and gender. If a child has BMI for age below the 5th percentile then it was considered as in the underweight group while a child with BMI for age above the 95th percentile was considered in the overweight group,^14^ with all other children classified as in the normal weight group.

Where information about medication intake was available, blood pressure readings were corrected for medication intake using published methods^15^ that assume that blood pressure from medicated individuals is higher than the observed measure. This correction was conducted by adding 15% of its value to the observed measure.

To correct for possible differences in the blood pressure measures taken in the first and second reading, we calculated the average of the two readings. Given the differences observed in blood pressure trajectories within the different studies, data from men and women were analysed separately.

## 3 Methods

Suppose we wish to model life course trajectories using splines in some way. As we will invariably have data on many individuals, we might consider fitting a separate spline to each individual and then characterising the population distribution of spline parameters in some way. However, this would require that all individuals share the same set of parameters, albeit with different values, which is impractical when individuals are observed over different stages of life, not least because each individual spline would have to be extrapolated into the unobserved age ranges. As an alternative, we propose using a single spline to define the population mean life course, with individuals’ departures from that mean behaviour accounted for by adding random effects to the spline rather than its parameters. We also add random effects to account for differences between cohorts/studies that are due to unmeasured covariates. A principal advantage of this approach is that the spline may be *adaptive*, with an a priori unknown number of knots. Individual-specific adaptive splines would have different numbers of parameters with different meanings, in general, which would preclude overall inferences.

### 3.1 Model description

Let $y_{\mathrm{ijk}}$ denote the *k*th blood pressure measurement $(k=1,\ldots ,K_{\mathrm{ij}})$ taken for the *j*th individual $(j=1,\ldots ,J_{i})$ in study *i* (i=1,…,I). We assume $y_{\mathrm{ijk}}\sim N(\mu_{\mathrm{ijk}},\sigma_{\mathrm{ijk}}^{2})$, with

$$\mu_{\mathrm{ijk}}=s_{\mathrm{ijk}}+\varphi_{i}+\gamma_{i}{\text{mrz}}_{\mathrm{ijk}}+\theta_{\mathrm{ij}}+r_{\mathrm{ijk}}$$

where $s_{\mathrm{ijk}}$ denotes the spline function to be fitted as described below; $\varphi_{i}$ and $\gamma_{i}$ are study-level random effects, representing study-specific deviations from the mean spline and effects of using an MRZ device, respectively (${\text{mrz}}_{\mathrm{ijk}}=1$ if $y_{\mathrm{ijk}}$ was measured using an MRZ device and ${\text{mrz}}_{\mathrm{ijk}}=0$ if an AO device was used), whilst $\theta_{\mathrm{ij}}$ represents an individual-level random effect. The term $r_{\mathrm{ijk}}$ represents the combined effects of various individual-level risk factors

$$r_{\mathrm{ijk}}=\alpha_{1_{i}}{\text{manual}}_{\mathrm{ijk}}+\alpha_{2_{i}}{\text{bmiu}}_{\mathrm{ijk}}+\alpha_{3_{i}}{\text{bmio}}_{\mathrm{ijk}}$$

In this expression parameters $\alpha_{1_{i}}$, $\alpha_{2_{i}}$ and $\alpha_{3_{i}}$ are study-level random effects and ${\text{manual}}_{\mathrm{ijk}}$, ${\text{bmiu}}_{\mathrm{ijk}}$ and ${\text{bmio}}_{\mathrm{ijk}}$ are binary indicators of manual occupation, being underweight and being overweight, respectively.

The spline *s_ijk_* is a piecewise polynomial given by

$$s_{\mathrm{ijk}}=\beta_{1}+\sum_{\ell =1}^{d}\beta_{\ell +1}(X_{\mathrm{ijk}}-x_{0})\ell +\sum_{l=1}^{q}\sum_{\ell =c}^{d}\beta_{\lambda (l,\ell )}(X_{\mathrm{ijk}}-\eta_{l})+\ell$$

for $X_{\mathrm{ijk}}\geq x_{0}$, where $X_{\mathrm{ijk}}$ denotes the age of individual *j* in study *i* at the *k*th observation time, *x*_0_ is the lowest age for which the function is defined and $x_{+}=x$ if x>0, $x_{+}=0$ otherwise. Parameters $\eta_{1},\ldots ,\eta_{q}$ are the ‘knots’ of the spline and the βs are regression coefficients. The constants c≥0 and d≥c represent the ‘continuity’ and ‘order’ of the spline, respectively, and λ(l,ℓ)=q+l×(d-c+1)+ℓ+1. In this paper we use a *linear* spline by selecting c=d=1. We choose a linear form, as opposed to a quadratic or cubic form, say, because then the knots correspond directly to change points in the fitted trajectory, whereas higher order splines can change direction away from the knots. This aids in interpreting the fitted trajectory and in specifying a prior distribution (see below) for the number of knots *q*, which is estimated as an unknown parameter, so that the smoothness of the fitted curve is estimated as part of the model, and hence the spline adapts itself to the information present in the data. We scale the age range so that the minimum and maximum values are 0 and 1, respectively.

Possible differences in the variability of measurements by device were accounted for by modelling the residual standard deviation $\sigma_{\mathrm{ijk}}$ as a function of the device used as follows

$$\sigma_{\mathrm{ijk}}=\sigma_{m_{i}}{\text{mrz}}_{\mathrm{ijk}}+\sigma_{a_{i}}(1-{\text{mrz}}_{\mathrm{ijk}})$$

where $\sigma_{a_{i}}$ and $\sigma_{m_{i}}$ are study-level random effects representing the study-specific residual standard deviations associated with the AO and MRZ devices, respectively.

### 3.2 Model estimation

The model was estimated within a Bayesian framework. Analyses were performed using WinBUGS^16,17^ with ‘Jump’ interface installed.^18^ As described by Lunn et al.,^18^ the reversible jump algorithm is implemented in WinBUGS^16,17^ to draw samples from the joint full conditional distribution of the coefficients β, knots η and number of knots *q*, whilst standard Gibbs/Metropolis steps are used to update the remaining model parameters. As the value of *q* changes during the Markov chain Monte Carlo (MCMC) simulation, so do the dimensions of β and η. One of the main challenges in such ‘variable dimension’ analyses is choosing sensible values for the spline coefficients when attempting a dimension-changing move. This is because the change of dimension necessitates a change of parameter space, which may have been visited previously (in the MCMC simulation) only rarely, if ever. Hence the MCMC sampler will have had little opportunity to learn about appropriate parameter values in the new space. This problem can be alleviated if we can derive the full conditional distribution for the coefficients in closed form, since appropriate coefficients can then be generated instantly for any set of proposed knots. A multivariate normal prior for the coefficients β combines with the normal likelihood to give a multivariate normal full conditional, which is straightforward to derive. In our analyses the prior mean and variance for each coefficient are 0 and 100^2^, respectively.

As the age range was scaled to the interval (0,1), we chose a Unif(0,1) distribution as a prior distribution for the location of each knot. The RJMCMC algorithm considered here increased the model flexibility with regard to the number of knots of the model. However, it requires a prior distribution for the number of knots *q*. We chose a Poisson prior with mean 3 as this distribution represents our a priori expectation that, after increasing throughout childhood and adolescence, blood pressure may begin to level off in early adulthood; it may then begin to increase again in middle age, with a possible further change in later life. The shape of the Poisson distribution then penalises large numbers of knots, which encourages parsimony.

The individual- and study-level random effects (or their logarithms) were assumed to arise from normal population distributions. For i=1,…, I

$$\theta_{\mathrm{ij}}\sim N(0,\zeta 2),\;j=1,\ldots ,J_{i},\varphi_{i}\sim N(0,\omega_{\varphi }^{2}),\gamma_{i}\sim N(m_{\gamma },\omega_{\gamma }^{2}),\alpha_{l_{i}}\sim N(m_{\alpha_{l}},\omega_{\alpha_{l}}^{2}),\;l=1,2,3,\log\sigma_{m_{i}}\sim N(m_{\sigma_{m}},\omega_{\sigma_{m}}^{2}),\log\sigma_{a_{i}}\sim N(m_{\sigma_{a}},\omega_{\sigma_{a}}^{2})$$

where the means and standard deviations are unknown parameters with appropriate, vague prior distributions

$$m_{\gamma },m_{\alpha_{1}},m_{\alpha_{2}},m_{\alpha_{3}}\sim \text{N}(0,1002),\;m_{\sigma_{m}},m_{\sigma_{a}}\sim \text{N}(\log10,1002)$$

$$\zeta ,\omega_{\varphi },\omega_{\gamma },\omega_{\alpha_{1}},\omega_{\alpha_{2}},\omega_{\alpha_{3}}\sim \text{Unif}(0,60),\;\omega_{\sigma_{m}},\omega_{\sigma_{a}}\sim \text{Unif}(0,10)$$

The upper bound of 60 for standard deviations defined on the natural scale is chosen as systolic pressures outside the range 0–240 mmHg are very unlikely or impossible. For standard deviations defined on the log-scale, an upper bound of 10 is chosen as this represents a vast degree of variability on the natural scale.

**Figure 1.**
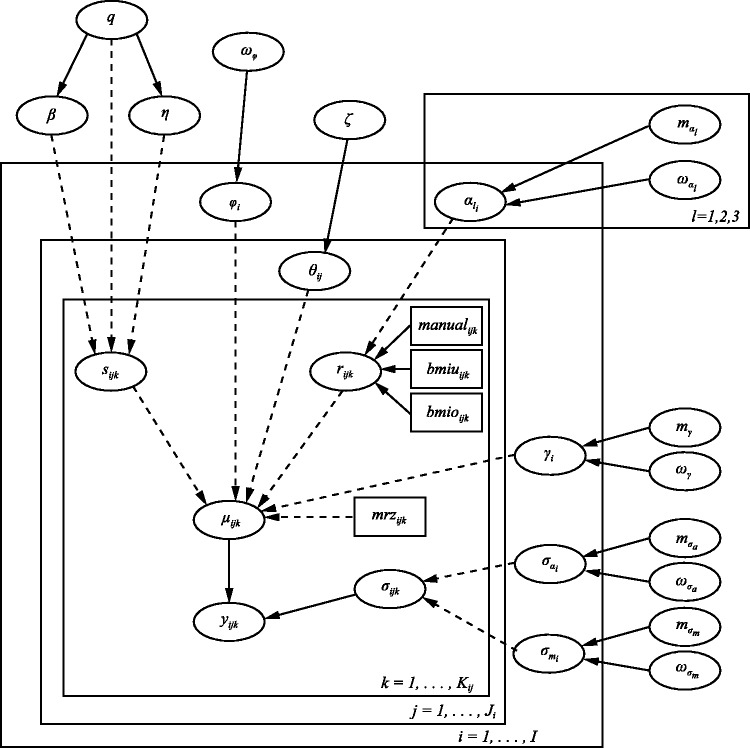
Directed acyclic graph (DAG) corresponding to blood pressure life course model. ‘Nodes’ represent variables in the model and are joined together by arrows to show direct dependence between variables. Solid arrows denote stochastic dependence whereas ‘dashed’ arrows denote logical dependence (deterministic functions). Rectangular nodes represent covariates that have been included in the model. The rectangular ‘containers’ labelled i=1,…,I, $j=1,\ldots ,J_{i}$, etc. denote repetition, i.e. ‘loops’ over the index used in the label.

The code can be found in Appendix 1, and a graphical representation of the model is presented in Figure 1. For each analysis, two MCMC chains were simulated from different initial states. Convergence was assessed via visual inspection of the trace plots and by the Brooks–Gelman–Rubin method.^19,20^ The Monte Carlo standard error (MCSE) for all parameters of interest was examined periodically to ensure accurate inferences. The rule of thumb of requiring an MCSE less than 5%^21^ (p. 277) was fully satisfied in our analysis, with <2% achieved for the vast majority of parameters.

## 4 Results

**Figure 2.**
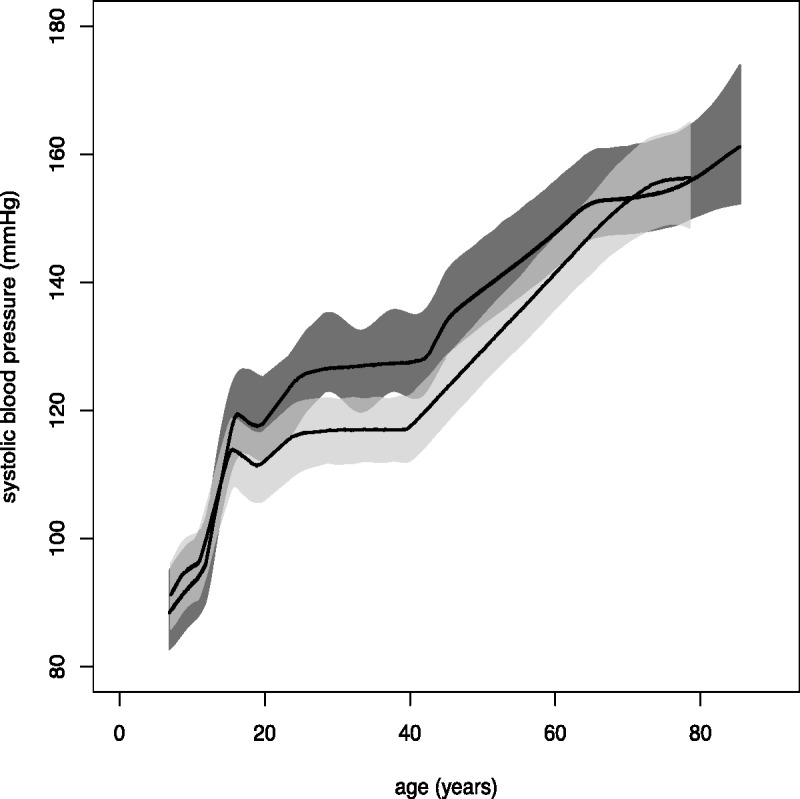
Overall systolic blood pressure trajectories, with 95% credible bands, for males (dark grey) and females (light grey).

Our results indicate that blood pressure increases with age as illustrated in Figure 2. The figure shows, for both genders, a rapid increase in blood pressure coinciding with peak adolescent growth, followed by a gentle increase in early adulthood, a midlife acceleration beginning in the fifth decade of life and a final period of deceleration in late adulthood. Following the rapid acceleration during adolescence, women typically have lower blood pressure than men (by up to around 10 mmHg), until their late 60s.

Table 2 presents posterior median estimates, along with 95% credible intervals, for the overall mean parameters. Male individuals with a manual occupation had higher blood pressure than individuals with a non-manual occupation (posterior median = 0.85). Although there is a suggestion that this is also the case for females (posterior median = 0.56), the effect is not statistically significant, with a posterior credible interval that includes zero. The effect of manual occupation on blood pressure seems to be greater for males. There is an indication that underweight individuals have lower blood pressure than normal weight individuals, but this effect is not significant for either gender. Overweight individuals, on the other hand, were found to have significantly higher blood pressure than normal weight individuals, for both males and females. For neither gender was there a significant effect on expected blood pressure of using an MRZ as opposed to an AO device. Posterior median parameter estimates for both genders were negative, however, suggesting that blood pressures may be lower, on average, when measured with an MRZ device. The level of residual variability was similar for both devices, suggesting that neither device is more variable than the other. Finally, a similar number of knots were estimated in modelling the men’s and women’s data, reflecting the fact that similar trajectories were estimated for both genders (see Figure 2). The estimated number of knots was also larger than expected a priori. This is probably due to confounding, leading to artefacts in the fitted spline, as discussed below.

**Table 2.** Posterior median estimates (with 95% credible intervals in parentheses) for overall parameters and inter-study/inter-individual standard deviations.

	Posterior median (95% credible interval)			
Risk factor/ Parameter	Women		Men	
	Overall	SD (inter-	Overall	SD (inter-
	mean	study/ind)	mean	study/ind)
Manual occupation ($\alpha_{1}$)	0.56 (−0.33, 1.65)	0.45 (0.02, 2.67)	0.85 (0.01, 1.61)	0.47 (0.03, 1.95)
Underweight ($\alpha_{2}$)	−2.22 (−7.61, 2.29)	3.19 (0.27, 12.91)	−2.86 (−6.53, 1.53)	2.76 (0.37, 10.22)
Overweight ($\alpha_{3}$)	4.87 (2.27, 7.72)	2.02 (0.79, 7.14)	5.15 (2.97, 7.51)	2.06 (0.98, 5.73)
Device (γ)	−1.04 (−3.94, 2.27)	1.00 (0.04, 7.94)	−3.48 (−12.95, 6.03)	7.46 (3.62, 24.32)
$\log\sigma_{m}$ (log-res sd(MRZ))	2.56 (1.69, 3.44)	0.50 (0.21, 2.52)	2.60 (2.09, 3.12)	0.41 (0.20, 1.35)
$\log\sigma_{a}$ (log-res sd(AO))	2.67 (1.91, 3.43)	0.60 (0.30, 2.00)	2.65 (2.08, 3.21)	0.54 (0.30, 1.44)
θ [individual-level RE]	0	7.07 (6.85, 7.29)	0	9.01 (8.75, 9.28)
φ [study-level RE]	0	5.10 (2.38, 15.92)	0	6.93 (3.49, 18.99)
Number knots (*q*)	10 (7, 13)	–	12 (9, 16)	–

Mean posterior estimates of study specific effects are presented in Table 3. The results suggest that individuals with a manual occupation have higher blood pressure than individuals with a non-manual occupation consistently over the different studies. However, in studies of female individuals, estimates only reached significance in ALSPAC, midlife T-07 cohort and NSHD. In studies of male individuals, significant estimates were found in midlife T-07, NSHD and CAPS studies. Underweight men and women typically had lower blood pressure than normal weight individuals. Although the direction of the effect reversed in older NSHD women, the effect of being underweight in this study did not reach significance (as it also did not for the older T-07 cohort, both genders, NSHD men and T-07 men in the midlife cohort). Consistently across studies, larger body size was significantly associated with higher blood pressure in both men and women.

**Table 3.** Posterior mean estimates, standard deviations and 95% credible intervals of study-specific parameters.

Parameter		Women		Men	
	Study	Mean (SD)	Cr. Interval	Mean (SD)	Cr. Interval
Mean effects					
$\alpha_{1}$ (Manual occupation)	ALSPAC	0.51 (0.22)	[0.07,0.95]	0.59 (0.30)	[−0.03,1.12]
	T-07 (1970s)	0.69 (0.46)	[−0.14,1.75]	0.58 (0.55)	[−0.71,1.49]
	T-07 (1950s)	1.05 (0.67)	[0.14,2.65]	1.08 (0.56)	[0.10,2.40]
	T-07 (1930s)	0.28 (0.62)	[−1.28,1.31]	0.65 (0.61)	[−0.82,1.73]
	NSHD	0.44 (0.19)	[0.06,0.80]	1.01 (0.21)	[0.62,1.43]
	CAPS	–	–	1.11 (0.44)	[0.36,2.11]
$\alpha_{2}$ (Underweight)	ALSPAC	−1.88 (0.37)	[−2.61,−1.15]	−2.17 (0.41)	[−2.98,−1.36]
	T-07 (1970s)	−2.65 (0.88)	[−4.41,−0.98]	−4.00 (0.95)	[−5.88,−2.20]
	T-07 (1950s)	−4.80 (2.39)	[−9.85,−0.90]	−2.33 (2.47)	[−7.08,3.07]
	T-07 (1930s)	−3.39 (2.90)	[−9.90,1.85]	−0.82 (3.35)	[−6.08,7.28]
	NSHD	0.85 (1.82)	[−2.37,4.44]	−1.67 (2.13)	[−5.47,2.99]
	CAPS	–	–	−5.75 (2.25)	[−10.47,−2.03]
$\alpha_{3}$ (Overweight)	ALSPAC	3.57 (0.20)	[3.19,3.96]	3.65 (0.23)	[3.21,4.09]
	T-07 (1970s)	4.92 (0.58)	[3.78,6.07]	5.93 (0.67)	[4.62,7.26]
	T-07 (1950s)	5.43 (0.66)	[4.14,6.75]	4.46 (0.73)	[3.02,5.88]
	T-07 (1930s)	7.12 (1.01)	[5.15,9.11]	7.27 (1.06)	[5.26,9.39]
	NSHD	3.52 (0.53)	[2.49,4.55]	3.32 (0.50)	[2.33,4.31]
	CAPS	–	–	6.45 (0.54)	[5.39,7.51]
φ (Study effect)	ALSPAC	6.49 (3.19)	[0.85,13.70]	8.37 (4.08)	[−3.01,15.53]
	T-07 (1970s)	−3.99 (2.83)	[−9.33,2.05]	−1.56 (3.78)	[−11.66,4.58]
	T-07 (1950s)	−0.96 (2.87)	[−6.69,5.00]	−1.22 (3.77)	[−11.22,4.91]
	T-07 (1930s)	0.37 (3.38)	[−6.54,7.05]	3.41 (3.87)	[−6.49,9.95]
	NSHD	−0.55 (2.87)	[−6.27,5.42]	−2.94 (3.77)	[−12.96,3.19]
	CAPS	–	–	−8.00 (3.85)	[−18.22,−1.76]
γ (Device)	ALSPAC	−0.98 (3.73)	[−6.80,5.02]	−3.49 (11.67)	[−26.48,19.56]
	T-07 (1970s)	−1.15 (1.52)	[−3.87,2.20]	−1.32 (2.46)	[−5.35,4.40]
	T-07 (1950s)	−1.31 (1.20)	[−3.66,1.09]	−4.69 (1.45)	[−7.54,−1.86]
	T-07 (1930s)	−0.69 (1.45)	[−3.38,2.40]	−10.38 (1.34)	[−13.02,−7.76]
	NSHD	−0.79 (1.13)	[−2.86,1.55]	−5.66 (1.59)	[−8.79,−2.63]
	CAPS	–	–	4.62 (1.11)	[2.44,6.80]
Variance effects					
$\sigma_{a}$ (AO device)	ALSPAC	7.04 (0.04)	[6.95,7.12]	6.75 (0.04)	[6.67,6.83]
	T-07 (1970s)	11.91 (0.36)	[11.23,12.63]	9.97 (0.35)	[9.30,10.67]
	T-07 (1950s)	17.35 (0.50)	[16.40,18.35]	16.16 (0.47)	[15.26,17.11]]
	T-07 (1930s)	22.33 (0.68)	[21.04,23.71]	21.01 (0.73)	[19.64,22.49]]
	NSHD	19.30 (0.40)	[18.53,20.10]	18.34 (0.39)	[17.59,19.12]
	CAPS	–	–	18.70 (0.55)	[17.66,19.80]
$\sigma_{m}$ (MRZ device)	T-07 (1970s)	9.15 (0.19)	[8.78,9.53]	9.83 (0.21)	[9.41,10.26]
	T-07 (1950s)	11.74 (0.27)	[11.22,12.29]	11.10 (0.27)	[10.59,11.65]
	T-07 (1930s)	20.53 (0.41)	[19.74,21.34]	18.74 (0.43)	[17.91,19.60]
	NSHD	12.91 (0.22)	[12.49,13.34]	11.42 (0.19)	[11.05,11.81]
	CAPS	–	–	19.00 (0.21)	[18.59,19.43]

It is interesting to note that the direction of estimated study-level random effects ($\varphi_{i}$) is consistently the same for both men and women, although only the ALSPAC effect for women and the CAPS effect for men are significantly non-zero. These suggest that ALSPAC women typically have blood pressures above the mean curve and CAPS men typically have blood pressures below the mean curve. Only in the CAPS study was the effect of using a different device (γ) significantly non-zero, indicating that measurement with the manual device was associated with higher blood pressures in the CAPS study. Study-specific estimates of residual variation were consistently similar for men and women, and for both devices. There appears to be an upward trend in residual variation with age.

Previous to the work reported here, we conducted a series of sensitivity analyses on each of the independent datasets to examine robustness of results to the method of adjustment for medication intake and to examine whether results varied if the first or second measure was modelled instead of their average. In both cases, results were robust.

Results reported here were obtained adjusting blood pressure measure for medication intake by adding 15% to the registered measure. To examine robustness of results to this method, we varied the percentage added (5, 10, 15, 20, 25%) and also added fixed constants (5, 10, 15 mmHg). Results obtained remained robust.

## 5 Discussion

In this paper, we propose a method to combine data from longitudinal studies of individuals in different stages of life to produce a life course trajectory. The method has been illustrated using blood pressure measures from four UK-based longitudinal studies. Our results elucidate the nature of the life course and provide scope for targeting public health interventions at specific age groups. They are also in general agreement with those reported by Wills et al., who found a rapid increase in blood pressure coinciding with peak adolescent growth, gentle increase in early adulthood, midlife acceleration beginning in the fifth decade of life and a final period of deceleration in late adulthood. In addition, they reported differences in blood pressure trajectories by gender, with a similar blood pressure level by the seventh decade. Our results show that following rapid acceleration during adolescence, women typically have lower blood pressure than men (by up to around 10 mmHg), until their late 60s. The local maxima occurring in the late teens for both genders are likely an artefact of the model rather than true maxima, as discussed below. Males with a manual occupation have higher blood pressures, as do overweight individuals from both genders. Although some cohort effects may exist on the risk factors that were not optimally considered when we opted for using cut-off points for the definition of BMI categories, our results suggest other interesting effects, such as underweight individuals having lower blood pressure and a trend between residual (unexplained) variation and age, but these have not been shown to be statistically significant.

Modelling life course trajectories is severely complicated by the fact that individuals are invariably only monitored over some fraction of their life course. In attempting to draw overall inferences about the population of interest, we might naturally wish to fit a parametric model to each individual’s data and characterise the population distribution of model parameters. However, unless the appropriate model is well established, this seems unrealistic, since in order to obtain a complete overall/population trajectory, each individual’s fitted trajectory must cover the whole life course, with much ‘borrowing of strength’ required to extrapolate into the (extensive) unobserved periods. If the parametric model is linear in the parameters, such as a spline, then one way around this might be to integrate out the individual-level parameters. However, this would require a bespoke MCMC algorithm, particularly if the number of knots is unknown, as here, rather than the more general modelling framework offered by BUGS, say. This might exploit some aspects of DiMatteo et al.’s RJMCMC approach, but would be substantially more complex due to the hierarchical structure of our model. (Note that we would have to assume a common set of knots for all individuals.) A very basic alternative might be to simply average the observed data in each of a series of short ‘age bins’, but this would preclude the possibility of controlling for covariates, say. Instead, our approach has been to start with the population life course trajectory and account for departures away from this ‘overall’ behaviour, due to variability between cohorts and individuals within cohorts, say, by adding random effects. Such an approach is limited, however, by the additive nature of the random effects. For example, excepting the effects of covariates, an individual can only deviate from his/her cohort’s overall trajectory, and that cohort can only deviate from the ‘global’ trajectory, by a constant amount (not changing with age). We could explore the inclusion of multiplicative random effects also, but the model’s flexibility would still be limited.

A principal objective of this work has been to avoid the need for strong parametric assumptions regarding the shape of the fitted trajectory whilst accommodating possible risk factors. By assuming the number and location of the spline knots to be unknown, the spline’s ‘smoothness’ is not pre-determined and it can adapt itself to the information in the data; estimation is thus largely data driven. As a consequence, the methodology is applicable to much smaller data sets than those considered herein, although the level of detail in the fitted curve will be reduced accordingly. We chose to use a *linear* adaptive spline, as opposed to a quadratic or cubic spline, say, because then the knots correspond directly to change points in the fitted trajectory, whereas higher order splines can change direction away from the knots (the change points also tend to be more ‘visible’ with a linear spline). This aids in the specification of a prior distribution for the number of knots and in the interpretation of the fitted trajectory. We note, however, that extensions to higher order splines are straightforward. In our work, a larger than initially expected number of knots was estimated (12 for men and 10 for women). This may be a consequence of the large amount of data included in the analysis, which allows even small variations in the data to be tracked inexpensively by the spline.

In our analyses, the estimation of study-level random effects ($\varphi_{i}$) presented some difficulty, with very high autocorrelation in the monitored Markov chains. This may be explained by several factors. First, there is limited overlap between some of the studies, which makes it difficult to identify differences between the studies. (In fact, in the presence of more overlap, in addition to a study effect, it may be necessary to include a cohort effect to better account for potential differences between the studies.) Second, there is a limited number of studies, meaning that even if contrasts between studies can be identified, the actual values of the random effects may still be poorly determined, since a wider range of values is supported by the hierarchical prior when the number of effects is small. Finally, the flexibility of the spline is such that, where there is little overlap, it can account for any differences between studies itself, and so the study effects and the spline are somewhat confounded, making for relatively slow exploration of the posterior distribution. The problem of slow exploration is alleviated straightforwardly by simulating longer Markov chains, though in our case this led to lengthy run-times, due to the large data sets (30–40,000 observations). The problem of confounding is much more challenging, and we believe may be responsible for the some of the ‘sudden’ direction changes in the fitted trajectories.

The introduced methodology assumes that missing information on blood pressure measurements is modelled as missing at random. Although a missing at random missing data assumption is likely to be plausible in the younger cohorts, it may not be realistic in the older cohorts as individuals with higher blood pressure are more likely to dropout of the studies. Extensions to the proposed method are under consideration to account for informative missing data.

## Appendix 1

model {

########## likelihood: ##########

for (k in 1:N) {

 sbp[k] ∼ dnorm(mu[k], inv.sigma2[k])

 mu[k] <- s[k] + phi[cohort[k]] + theta[id[k]] + gamma[cohort[k]]*mrz[k] + r[k]

 inv.sigma2[k] <- 1/pow(sigma[k], 2)

 sigma[k] <- sigma.a[cohort[k]]*a[k] + sigma.m[cohort[k]]*mrz[k]

 r[k] <- alpha[cohort[k], 1]*manual[k] + alpha[cohort[k], 2]*bmiu[k] + alpha[cohort[k], 3]*bmio[k]

 scaled.age[k] <- (X[k] - min.age)/(max.age - min.age)

}

s[1:N] <- jump.pw.poly.cs.lin(scaled.age[1:N], q, 0.0001, 0, 1, inits[])

########## random effects: ##########

for (i in 1:I) {

 phi[i] ∼ dnorm(0, tau.phi)

 for (j in 1:3) {alpha[i, j] ∼ dnorm(m.alpha[j], tau.alpha[j])}

 gamma[i] ∼ dnorm(m.gamma, tau.gamma)

 log(sigma.a[i]) <- log.sigma.a[i]

 log(sigma.m[i]) <- log.sigma.m[i]

 log.sigma.a[i] ∼ dnorm(m.sigma.a, tau.sigma.a)

 log.sigma.m[i] ∼ dnorm(m.sigma.m, tau.sigma.m)

}

for (j in 1:num.ind) {theta[j] ∼ dnorm(0, inv.zeta2)}

########## priors: ##########

q ∼ dpois(3)I(, 50)

for (j in 1:3) {

 m.alpha[j] ∼ dnorm(0, 0.0001)

 tau.alpha[j] <- 1/pow(omega.alpha[j], 2)

 omega.alpha[j] ∼ dunif(0, 60)

}

 m.gamma ∼ dnorm(0, 0.0001)

 m.sigma.a ∼ dnorm(2.303, 0.0001)

 m.sigma.m ∼ dnorm(2.303, 0.0001)

 tau.gamma <- 1/pow(omega.gamma, 2)

 tau.sigma.a <- 1/pow(omega.sigma.a, 2)

 tau.sigma.m <- 1/pow(omega.sigma.m, 2)

 tau.phi <- 1/pow(omega.phi, 2)

 inv.zeta2 <- 1/pow(zeta, 2)

 omega.gamma ∼ dunif(0, 60)

 omega.sigma.a ∼ dunif(0, 10)

 omega.sigma.m ∼ dunif(0, 10)

 omega.phi ∼ dunif(0, 60)

 zeta ∼ dunif(0, 60)

}

}

## References

[1] Laird NM Ware JH Random-effects models for longitudinal data. Biometrics 1982; 38: 963–974. 7168798

[2] Lieb W Xanthakis V Sullivan LM Longitudinal tracking of left ventricular mass over the adult life course: Clinical correlates of short- and long-term change in the Framingham Offspring Study. Circulation 2009; 119: 3085–3092. 19506113 10.1161/CIRCULATIONAHA.108.824243 PMC2761217

[3] Cheng S Xanthakis V Sullivan LM Correlates of echocardiographic indices of cardiac remodeling over the adult life course longitudinal observations from the Framingham Heart Study. Circulation 2010; 122: 570–578. 20660804 10.1161/CIRCULATIONAHA.110.937821 PMC2942081

[4] Stewart R Xue QL Masaki K Change in blood pressure and incident dementia: A 32-year prospective study. Hypertension 2009; 54: 233–240. 19564551 10.1161/HYPERTENSIONAHA.109.128744 PMC3136040

[5] Hastie TJ Tibshirani RJ Generalized additive models, London: Chapman & Hall, 1990.

[6] DiMatteo I Genovese C Kass R Bayesian curve-fitting with free-knot splines. Biometrika 2001; 88: 1055–1071.

[7] Green PJ Reversible jump Markov chain Monte Carlo computation and Bayesian model determination. Biometrika 1995; 82: 711–732.

[8] Wills A Lawlor DA Matthews F Lifecourse trajectories of systolic blood pressure using longitudinal data from eight UK cohorts. PLos Med 2011; 6: e10000440–e10000440. 10.1371/journal.pmed.1000440 PMC3114857 21695075

[9] Golding J The Avon Longitudinal Study of Parents and Children (ALSPAC) – study design and collaborative opportunities. Eur J Endocrinol 2004; 151: U119–U123. 15554896 10.1530/eje.0.151u119

[10] Wadsworth M Richards D Hardy R Cohort profile: The 1946 National Birth Cohort (MRC National Survey of Health and Development). Int J Epidemiol 2006; 35: 49–54. 16204333 10.1093/ije/dyi201

[11] Caerphilly and Speedwell Collaborative Heart Disease Studies. The Caerphilly and Speedwell Collaborative Group. J Epidemiol Community Health 1984; 38: 259–262. 6332166 10.1136/jech.38.3.259 PMC1052363

[12] Benzeval M Der G Ellaway A Cohort profile: West of Scotland Twenty-07 study: Health in the community. Int J Epidemiol 2009; 38: 1215–1223. 18930962 10.1093/ije/dyn213 PMC2935558

[13] Expert Committee on Physical Status W. *The use and interpretation of antropometry*. Geneva: World Health Organisation, 1993.

[14] Rosner B Prineas R Loggie J Percentiles for body mass index in U.S. children 5 to 17 years of age. J Pediatr 1998; 132: 211–222. 9506630 10.1016/s0022-3476(98)70434-2

[15] Tobin MD Sheehan NA Scurrah KJ Adjusting for treatment effects in studies of quantitative traits: Antihypertensive therapy and systolic blood pressure. Stat Med 2005; 24: 2911–2935. 16152135 10.1002/sim.2165

[16] Lunn DJ Thomas A Best N WinBUGS – a Bayesian modelling framework: Concepts, structure, and extensibility. Stat Comput 2000; 10: 325–337.

[17] Lunn D Spiegelhalter D Thomas A The BUGS project: Evolution, critique and future directions (with discussion). Stat Med 2009; 28: 3049–3082. 19630097 10.1002/sim.3680

[18] Lunn DJ Best N Whittaker JC Generic reversible jump MCMC using graphical models. Stat Comput 2009; 19: 395–408.

[19] Gelman A Rubin DB Inference from iterative simulation using multiple sequences (with discussion). Stat Sci 1992; 7: 457–511.

[20] Brooks SP Gelman A General methods for monitoring convergence of iterative simulations. J Comput Graph Stat 1998; 7: 434–455.

[21] Gelman A Carlin JB Stern HS Bayesian data analysis, 2nd ed London: Chapman and Hall/CRC, 2004.

